# Digitalisierung im Gesundheitswesen zur Stärkung der Patient:innenautonomie

**DOI:** 10.1007/s00103-025-04181-8

**Published:** 2026-01-09

**Authors:** Mona Rams, Stefanie Rudolph, Petya Zyumbileva, Claudia Grehn, Tamara Hussong Milagre, Nadja Will, Christof von Kalle

**Affiliations:** 1 Clinical Study Center, Berlin Institute of Health (BIH) at Charité – Universitätsmedizin Berlin, Charitéplatz 1, 10117 Berlin, Deutschland; 2 Patientenorganisation EVITA, Lissabon, Portugal; 3 Bielefeld, Nordrhein-Westfalen Deutschland; 4 Luxembourg Research Clinic (LRC) – Fuerschungsklinik Lëtzebuerg, Luxemburg Institute of Health, Luxembourg, Luxemburg

**Keywords:** Digitale Selbstbestimmung, Gesundheitsdatennutzung, Teilhabe im Gesundheitssystem, Gesundheitspolitische Rahmenbedingungen, Digitale Technologien, Digital self-determination, Health data use, Participatory healthcare, Health policy framework, Digital technologies

## Abstract

Patient:innenautonomie gilt als zentrales ethisches und gesundheitspolitisches Leitprinzip moderner Versorgung. Ihre praktische Ausgestaltung gewinnt angesichts der rasch voranschreitenden digitalen Transformation des Gesundheitswesens zunehmend an Bedeutung. Digitale Technologien verändern die Bedingungen, unter denen Autonomie ausgeübt und gesichert werden kann. Sie verändern den Zugang zu Informationen und die Orientierung im Versorgungsgeschehen und haben damit Einfluss auf individuelle Entscheidungsprozesse.

Vor dem Hintergrund dieser Entwicklungen erweist sich ein rein individualistisch verstandenes Autonomiekonzept als unzureichend. Soll die Digitalisierung des Gesundheitswesens tatsächlich zur Stärkung der Patient:innenautonomie beitragen, muss über die technische und organisatorische Komponente hinaus Autonomie konzeptionell mitgedacht und strukturell berücksichtigt werden. Daraus ergibt sich ein politisch- und sozialstruktureller Gestaltungsauftrag, der weit über die Einführung einzelner Anwendungen hinausreicht.

Die konsequente Berücksichtigung eines erweiterten Autonomieverständnisses stellt hohe Anforderungen an digitale Infrastrukturen und deren systematische Einbettung. Digitale Lösungen müssen verlässlich funktionieren, gesellschaftliche Vielfalt berücksichtigen, Teilhabe ermöglichen und Orientierung fördern. Um informationelle und partizipative Selbstbestimmung abzusichern, ist zudem ein Zusammenspiel zielgerichteter kohärenter Regulierung, politischer Steuerung und sozialsensibler struktureller Bedingungen erforderlich. Ziel des Beitrags ist es, diese Anforderungen differenziert herauszuarbeiten und sie mit Blick auf eine patient:innenorientierte Ausgestaltung digitaler Infrastrukturen als emanzipatorischen Gestaltungsauftrag zu profilieren.

## Abstract (en)

Patient autonomy is regarded as a central ethical and health policy principle of modern healthcare delivery. Its practical implementation is gaining increasing importance in light of the rapidly advancing digital transformation of the healthcare system. Digital technologies are changing the conditions under which autonomy can be exercised and safeguarded. They alter access to information and orientation within healthcare settings, thereby influencing individual decision-making processes. Against this backdrop, a purely individualistic concept of autonomy proves insufficient. For the digitalization of healthcare to meaningfully strengthen patient autonomy, it must go beyond technical and organisational implementation and include a conceptual and structural engagement with autonomy. This gives rise to a political and socio-structural design mandate that goes far beyond the introduction of individual applications. A consistent commitment to an expanded understanding of autonomy places substantial demands on the development and integration of digital infrastructures. Digital solutions must function reliably, reflect societal diversity, enable participation and support orientation. Safeguarding informational and participatory self-determination additionally requires an interplay of targeted political governance, coherent regulation, technically robust interoperability and socially responsive structural conditions. The aim of this article is to examine these requirements in detail and to profile them, with regard to a patient-oriented design of digital infrastructures, as an emancipatory mandate for a patient-centered health care system.

## Einleitung

Patient:innenautonomie ist ein zentrales ethisches, rechtliches und zunehmend auch gesundheitspolitisches Leitprinzip der modernen Medizin. Sie beschreibt das Recht und die Fähigkeit von Patient:innen, auf der Basis hinreichender Informationen selbstbestimmte Entscheidungen über diagnostische, therapeutische und präventive Maßnahmen zu treffen [1]. In ihrer traditionellen Form ist sie stark an die Ärzt:innen-Patient:innen-Beziehung und deren kommunikative Qualität gebunden [2]. Der zunehmende Einsatz digitaler Technologien verändert die Bedingungen der Patient:innenautonomie grundlegend, etwa durch erweiterten Zugang zu Gesundheitsinformationen, neue Formen der Nachvollziehbarkeit von Entscheidungen und Möglichkeiten zur Mitgestaltung der Versorgung. Damit wird Patient:innenautonomie im digitalen Zeitalter zu einem vielschichtigen Phänomen, geprägt von individuellen, sozialstrukturellen, technischen und regulatorischen Ebenen [3].

Die Digitalisierung des Gesundheitswesens, die auch international stark voranschreitet [4], bietet die Möglichkeit, Patient:innen nicht nur als Teil der Versorgung, sondern als aktiv Mitgestaltende einzubinden. Entscheidungen beruhen zunehmend auf digitalen Datenflüssen, Schnittstellen und technischen Anwendungen, die sich oft der individuellen Kontrolle entziehen [3, 5]. Während digitale Technologien politisch als Treiber für Effizienz, Transparenz und Qualität gelten, wurden ihr Beitrag zur Stärkung der Patient:innenautonomie sowie die dafür notwendigen strukturellen Voraussetzungen bislang kaum systematisch analysiert [6].

Digitalisierungsprozesse beeinflussen unterschiedliche Ebenen des Gesundheitssystems. Auf der Makroebene wirken gesundheitspolitische Entscheidungen, rechtliche Rahmenbedingungen und Finanzierungsmodelle als strukturgebende Kräfte der digitalen Transformation. Auf der Mesoebene gestalten Organisationen wie Krankenhäuser, Krankenkassen oder Gesundheitsplattformen die institutionelle Umsetzung digitaler Prozesse. Auf der Mikroebene stehen individuelle Patient:innen und Leistungserbringende im Zentrum, etwa bei der Nutzung von Gesundheits-Apps, der Einsicht in die elektronische Patientenakte (ePA) oder der Teilnahme an Telekonsultationen [7, 8].

Damit Digitalisierung Patient:innenautonomie stärkt, müssen technologische Lösungen und regulatorische Rahmenbedingungen gezielt auf Mitsprache, Kontrolle und Teilhabe ausgerichtet sein. Dazu gehören barrierefreier Informationszugang, digitale Gesundheitskompetenz, Strukturen für informierte Entscheidungen, Einbindung in Behandlungsprozesse und eine verlässliche Systemnavigation. Die Digitalisierung eröffnet die Möglichkeit, Patient:innenautonomie als sozial eingebettetes und strukturell verankertes Prinzip neu zu gestalten [8, 9].

Der Beitrag zeigt, wie digitale Gesundheitslösungen Patient:innenautonomie verändern und erweitern. Leitend ist die Frage, unter welchen Bedingungen sie Autonomie fördern oder behindern. Dazu werden Anwendungsfelder, normative Konzepte sowie sozialstrukturelle und politisch-regulatorische Rahmenbedingungen analysiert. Ziel ist es, einen umfassenden Überblick zu liefern und die Digitalisierung als emanzipatorischen Gestaltungsauftrag für ein patient:innenzentriertes Gesundheitssystem zu profilieren.

## Digitale Gesundheitslösungen mit Autonomiepotenzial

**Abb. 1:**
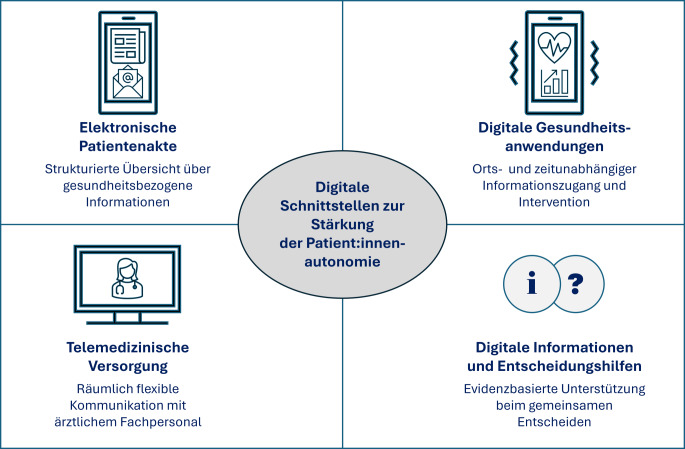
Digitale Gesundheitslösungen zur Stärkung der Patient:innenautonomie: 4 Anwendungsfelder, die exemplarisch Schnittstellen zwischen technologischer Infrastruktur und Versorgungspraxis markieren. (Eigene Abbildung erstellt mithilfe von ChatGPT4o)

Autonomie im digitalen Gesundheitswesen wird insbesondere dort relevant, wo technische Infrastrukturen Entscheidungen und Interaktionen in der Versorgung prägen. Bevor auf normative, sozialstrukturelle und politische Faktoren eingegangen wird, werden 4 Anwendungsfelder exemplarisch beleuchtet, die Schnittstellen zwischen technologischer Infrastruktur und Versorgungspraxis markieren: ePA, digitale Gesundheitsanwendungen (DiGA), telemedizinische Angebote und digitale Gesundheitsinformationen (Abb. 1). Diese Anwendungen adressieren zentrale Dimensionen von Patient:innenautonomie, sind bereits regulatorisch verankert und zeigen exemplarisch Potenziale und Herausforderungen der digitalen Transformation im Versorgungskontext.

### Elektronische Patientenakte

Die ePA ermöglicht Patient:innen und behandelnden Gesundheitsfachpersonen eine strukturierte und sektorenübergreifende Übersicht über Gesundheitsdaten, wie Medikationspläne, Arztbriefe, Impfstatus und Notfalldaten [10]. Mit dem Opt-out-Verfahren in Deutschland werden eine automatische Bereitstellung der ePA für gesetzlich Versicherte eingeführt und eine breite Nutzung angestrebt [11]. Dadurch sollen medizinische Informationen von der Vorsorge über die Therapie bis hin zur Nachsorge einrichtungs- und sektorenübergreifend verfügbar sein [10, 12].

Ein zentrales Autonomiepotenzial der ePA liegt in der individuellen Steuerung des Datenzugriffs. Patient:innen können festlegen, welche Gesundheitsfachpersonen welche Informationen sehen dürfen. Zudem stärkt die ePA die Transparenz über den eigenen Krankheitsverlauf und unterstützt informierte Teilhabe an diagnostischen und therapeutischen Entscheidungen [13]. Dadurch ermöglicht sie eine aktivere Rolle der Patient:innen im Versorgungsgeschehen.

Im internationalen Vergleich ist die Nutzung und Akzeptanz von ePA bereits deutlich weiter verbreitet, z. B. in Estland, Dänemark, Israel und Österreich [4, 14, 15]. In Deutschland hingegen ist die sie bislang noch begrenzt akzeptiert, bedingt durch zögerliche Nutzung, anhaltende Datenschutzbedenken und einen teils unklaren Mehrwert [16]. Erfolgsfaktoren sind konsequente Digitalstrategien, eine verständliche Nutzeroberfläche, einheitliche Datenformate, klare datenschutzrechtliche Rahmenbedingungen sowie eine hohe gesellschaftliche Akzeptanz [17].

### Digitale Gesundheitsanwendungen

Digitale Gesundheitsanwendungen (DiGA) eröffnen Patient:innen neue Wege der Selbstbeobachtung, Intervention und Informationsvermittlung. Seit der Einführung des sog. Fast-Track-Verfahrens durch das Digitale-Versorgung-Gesetz im Jahr 2019 können digitale Medizinprodukte niedriger Risikoklasse durch das Bundesinstitut für Arzneimittel und Medizinprodukte (BfArM) geprüft und bei positiver Bewertung in das offizielle DiGA-Verzeichnis aufgenommen werden [18]. Gesetzlich Versicherte haben seither einen Anspruch auf Erstattung, sofern eine ärztliche Verordnung oder eine Genehmigung durch die Krankenkasse vorliegt [19, 20].

Im Hinblick auf Patient:innenautonomie fördern DiGA orts- und zeitunabhängigen Zugang zu gesundheitsbezogenen Informationen, die selbstständige Auseinandersetzung mit dem eigenen Gesundheitszustand und stärken eine aktive Rolle im Versorgungsprozess. Durch Symptomtagebücher, interaktive Module oder personalisierte Rückmeldungen können Patient:innen ihre Krankheitsverläufe nachvollziehen und Behandlungen mitgestalten. Studien zeigten, dass DiGA das Autonomieempfinden steigern können, insbesondere bei psychischen Erkrankungen, Schmerzstörungen oder chronischen Leiden [19, 21, 22].

### Telemedizinische Versorgung

Telemedizin erweitert ärztliche Leistungen durch orts- und zeitunabhängige Kommunikationsformen wie Videosprechstunden oder asynchrone Konsultationen. Dies kann den Versorgungszugang insbesondere für mobilitätseingeschränkte, chronisch erkrankte oder ländlich wohnende Patient:innen deutlich verbessern [23].

Darüber hinaus kann Telemedizin Gesundheitskompetenz und Mitgestaltung fördern. Digitale Kommunikationsformate ermöglichen Patient:innen, sich gezielter über Krankheitsverläufe, Behandlungsoptionen und ärztliche Empfehlungen zu informieren und Rückfragen niedrigschwelliger zu stellen. So erweitert Telemedizin nicht nur den Zugang, sondern auch die informierte Teilhabe [23, 24].

### Digitale Gesundheitsinformationen

Der Zugang zu qualitätsgesicherten digitalen Gesundheitsinformationen ist eine zentrale Voraussetzung für informierte Entscheidungen und damit ein Kernelement patient:innenzentrierter Versorgung. Evidenzbasierte Gesundheitsportale, Entscheidungshilfen oder strukturierte Videoformate können dazu beitragen, Gesundheitskompetenz zu fördern und Patient:innen in ihrer Rolle als mitentscheidende Akteur:innen zu stärken [24].

Digitale Gesundheitsinformationen bieten großes Potenzial, da sie niedrigschwellig verfügbar sind, eine breite Reichweite entfalten und Patient:innen orts- und zeitunabhängig unterstützen können. Zugleich bergen sie im Unterschied zu ePA, DiGA und Telemedizin besondere Risiken, da Qualität und Verlässlichkeit nicht institutionell abgesichert sind. Eine unüberschaubare Informationsfülle, widersprüchliche Aussagen und kommerzielle Interessen können zudem zu Unsicherheit oder Fehlentscheidungen führen. Die Etablierung vertrauenswürdiger, zielgruppenspezifischer Informationsangebote ist ein zentraler Baustein digitaler Autonomieförderung [25, 26].

## Normative Konzepte einer emanzipatorischen Digitalisierung

Im Rahmen einer patient:innenorientierten digitalen Transformation gewinnen Konzepte an Bedeutung, die digitale Selbstbestimmung rechtlich und regulatorisch verankern. Dazu zählen Datenschutz, Datennutzung und Governance als autonomieförderliche Rahmenbedingungen [27, 28].

**Abb. 2:**
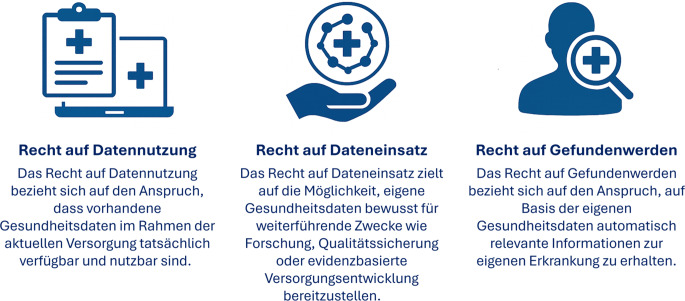
Drei Kernrechte der Patient:innen im Umgang mit Gesundheitsdaten als Grundlage digitaler Selbstbestimmung. (Eigene Abbildung erstellt mithilfe von ChatGPT4o)

Aufbauend auf dem praktischen Bedarf an patient:innenzentrierten Regelungen lassen sich 3 Kernrechte formulieren, die als Leitplanken einer emanzipatorischen Digitalisierung gelten sollten (Abb. 2; [29–31]). Diese umfassen nicht nur die Einsichtnahme oder passive Bereitstellung von Informationen, sondern aktive Gestaltungs- und Teilhabemöglichkeiten:

### Recht auf Datennutzung.

Das Recht auf Datennutzung bezieht sich auf den Anspruch, dass vorhandene Gesundheitsdaten im Rahmen der aktuellen Versorgung verfügbar und nutzbar sind, etwa durch interoperable ePA, sektorübergreifende Informationsflüsse und die technische Lesbarkeit für Behandelnde. Entscheidend ist die konkrete Verwendbarkeit im klinischen Alltag, nicht die bloße Existenz digitaler Dokumente.

### Recht auf Dateneinsatz.

Das Recht auf Dateneinsatz zielt auf die Möglichkeit, eigene Gesundheitsdaten bewusst für weiterführende Zwecke wie Forschung, Qualitätssicherung oder evidenzbasierte Versorgungsentwicklung bereitzustellen. Voraussetzung sind verständliche, niedrigschwellige Informationen über Formen, Ziele und Schutzmechanismen der Datennutzung. Die Datenbereitstellung wird so zu einem Ausdruck informierter Mitwirkung und solidarischer Verantwortung. Auf dieser Basis entsteht ein lernendes Gesundheitssystem, das reale Versorgungsdaten einbezieht, wissenschaftliche Erkenntnisse generiert und langfristig Qualität, Sicherheit und Gerechtigkeit stärkt.

### Recht auf Gefundenwerden.

Das Recht auf Gefundenwerden bezieht sich auf den Anspruch, auf Basis der eigenen Gesundheitsdaten proaktiv mit relevanten Informationen in Verbindung gebracht zu werden, etwa zu geeigneten Therapiemöglichkeiten, passenden Studien, Forschungsvorhaben oder Versorgungsangeboten. So wird ein gleichberechtigter Zugang zu innovativer Versorgung und Teilhabe gefördert, unabhängig von Wohnort, sozialem Status oder institutionellen Zufällen.

Diese 3 Rechte tragen aktiv zur Stärkung der Patient:innenautonomie bei, indem sie Daten als Grundlage aktiver Teilhabe verstehen. Sie betonen nicht nur den Schutz sensibler Daten, sondern auch die Potenziale einer souveränen Datennutzung im Sinne von Patient:innen.

Diese Teilhaberechte zeigen, dass Datenschutz über reine Schutzfunktionen hinausgedacht werden muss. Aktuell wird er häufig im Sinne eines defensiven Abwehrrechts interpretiert und birgt die Gefahr, dass Potenziale für Versorgung, Forschung und Prävention ungenutzt bleiben. Für die Stärkung der Patient:innenautonomie ist ein zukunftsgerichteter Datenschutz erforderlich, der Schutz und verantwortliche Nutzbarkeit verbindet [32, 33].

*Aus Patient:innenperspektive* ist klar: Digitalisierung muss spürbare Verbesserungen im Alltag bringen. Frust entsteht, wenn trotz großer Datenmengen keine passenden Informationen oder Behandlungsmöglichkeiten auffindbar sind, was im Ernstfall entscheidend für den Krankheitsverlauf ist. Patient:innen wollen nicht nur informiert, sondern beteiligt sein: Sie sollen das Recht auf Datennutzung, Dateneinsatz und Gefundenwerden aktiv wahrnehmen können.

Interoperable, vertrauenswürdige Systeme sowie Aufklärung über Datenumgang fördern Autonomie und Teilhabe. Die Digitalisierung eröffnet die Chance auf eine ermächtigende, gut informierte Zukunft, in der künstliche-intelligenzbasierte (KI-gestützte) Systeme die Patient:innenautonomie durch personalisierte Prävention, Früherkennung und individuelle Gesundheitsstrategien stärken. Digitalisierung darf keine zusätzliche Belastung sein, sie muss sich an realen Bedürfnissen orientieren und Versorgung sowie Forschung sinnvoll, praxisnah und gerecht unterstützen.

Ein moderner Datenschutz muss gewährleisten, dass Gesundheitsdaten gezielt, sicher und patient:innenorientiert verwendet werden können. Wird er als Ermöglichungsrahmen gedacht, kann er Teilhabe, Vertrauen und Qualität einer lernenden, digitalen Versorgung fördern [32].

## Sozialstrukturelle Rahmenbedingungen für Autonomiegewinn

Digitale Technologien entfalten ihr Autonomiepotenzial nicht allein durch ihre funktionalen Eigenschaften, sondern auch durch die soziokulturellen und strukturellen Bedingungen, unter denen sie genutzt werden. Während Entscheidungen zunehmend auf digitalen Datenflüssen, Schnittstellen und technischen Anwendungen beruhen, entziehen sich diese der individuellen Kontrolle [3, 5]. Im digitalen Kontext greift daher ein rein individualistisch verstandenes Autonomiekonzept zu kurz. Der Zugang zu digitalen Anwendungen sowie ihre sinnvolle Nutzung setzen neben individuellen auch gesellschaftlichen Bedingungen voraus, die im Sinne gesundheitlicher Chancengleichheit aktiv gestaltet werden müssen.

Im Rahmen der sozialstrukturellen Faktoren für die Stärkung der Patient:innenautonomie ist die individuelle digitale Gesundheitskompetenz entscheidend. Sie umfasst die Fähigkeit, gesundheitsbezogene Informationen in digitalen Umgebungen zu finden, zu verstehen, zu bewerten und anzuwenden. Studien ergaben, dass ein erheblicher Teil der Bevölkerung hierbei auf Schwierigkeiten stößt [34]. Dies kann negative Konsequenzen für Autonomie, Versorgungsteilnahme und Therapieadhärenz haben [24].

Neben bildungspolitischen Maßnahmen ist daher auch niedrigschwellige Unterstützung im Versorgungsalltag erforderlich: Lots:innen, digitale Gesundheitsberater:innen oder andere vermittelnde Instanzen. Überdies ist es unerlässlich, neben der individuellen auch die institutionelle Dimension digitaler Gesundheitskompetenz aktiv zu gestalten, was sowohl die Qualifizierung von Personal als auch die Neugestaltung von Strukturen und Prozessen im Gesundheitswesen umfasst.

Technische Barrieren, komplexe Nutzeroberflächen oder fehlende Interoperabilität können die selbstbestimmte Nutzung digitaler Gesundheitsangebote erheblich einschränken. Um Patient:innenautonomie zu fördern, sollten Anwendungen benutzerzentriert gestaltet sein. Dazu zählen Verständlichkeit, intuitive Navigation, Barrierefreiheit und sprachliche Diversität. Dies gilt insbesondere für vulnerable Gruppen wie ältere Menschen, Menschen mit Behinderung oder Personen mit Migrationshintergrund [9].

Auch der demografische Wandel ist im Kontext der Digitalisierung des Gesundheitswesens zu berücksichtigen. Angesichts zunehmender Morbidität und eines wachsenden Fachkräftemangels steht das Gesundheitssystem vor neuen Anforderungen. Auf Seite der Leistungserbringenden kann zudem insbesondere für ältere Kolleg:innen der Umgang mit digitalen Entwicklungen herausfordernd sein. Da die aktive Begleitung durch die Ärzteschaft und weitere Heilberufe unabdingbar ist, um die Potenziale der Digitalisierung zu erschließen, sind gezielte Fortbildungen und praxisnahe Unterstützungsangebote erforderlich. Nicht zuletzt kann die Digitalisierung, richtig verankert, gerade im Kontext des demografischen Wandels zur Entlastung des Gesundheitssystems beitragen.

Im Kontext sozialstruktureller Voraussetzungen für digitale Teilhabe rückt auch die Literalität in den Blick. In Deutschland verfügen rund 6,2 Mio. Erwachsene lediglich über eingeschränkte Lese- und Schreibkompetenzen [35]. Während textbasierte Informationen den Zugang für Betroffene erschweren und damit Unsicherheiten oder Fehlentscheidungen begünstigen können, eröffnen digitale Lösungen durch multimediale Ansätze wie audiovisuelle Inhalte oder interaktive Darstellungen erweiterte Möglichkeiten der Informationsvermittlung. Entscheidend ist eine Gestaltung, die Barrieren berücksichtigt und zugleich Potenziale nutzt, um Autonomie und Teilhabe auch für Menschen mit eingeschränkter Literalität zu fördern.

Eine weitere Voraussetzung für die Stärkung der Patient:innenautonomie im digitalen Wandel ist die kulturelle Passung digitaler Angebote im Sinne einer umfassenden digitalen Inklusion. Nur wenn Technologien an die sprachlichen, sozialen und lebensweltlichen Hintergründe ihrer Anwender:innen anschließen, können sie Vertrauen schaffen und zur selbstbestimmten Nutzung beitragen. Andernfalls besteht das Risiko, dass digitale Angebote an den Erfahrungen vieler Patient:innen vorbeigehen [9].

Wenn digitale Strukturen zunehmend zur Voraussetzung für Zugang, Information oder Rückmeldung werden, besteht das Risiko einer stillen Exklusion all jener, die aufgrund technischer, sprachlicher, kultureller oder psychosozialer Barrieren nicht gleichermaßen partizipieren können. Wird die Ausweitung digitaler Möglichkeiten zur Selbstbestimmung inkonsequent implementiert, kann sie paradoxerweise dazu führen, dass bestehende Ungleichheiten nicht abgebaut, sondern weiter vertieft werden [36].

*Aus Patient:innenperspektive* greift eine rein technikzentrierte Digitalisierung zu kurz. Digitale Gesundheitsangebote entfalten ihren Wert nur, wenn sie verständlich, niedrigschwellig zugänglich und sinnvoll in die reale Versorgung eingebettet sind. Patient:innen benötigen Unterstützung, um digitale Informationen einordnen, Risiken bewerten und geeignete Anwendungen finden zu können. Die Förderung digitaler Gesundheitskompetenz ist dabei zentral. Nutzerfreundlichkeit und kulturelle Passung digitaler Lösungen schaffen Vertrauen und ermöglichen gerechte Teilhabe. Es darf nicht Aufgabe der Patient:innen sein, sich allein durch unübersichtliche Informationen zu kämpfen.

Patient:innenautonomie im digitalen Wandel erfordert nicht nur Zugang zu Technologien, sondern auch die Fähigkeit, diese selbstbestimmt und im Einklang mit individuellen Lebenslagen zu nutzen. Dafür wird ein Gesundheitssystem benötigt, das soziale Unterschiede mitdenkt und strukturelle Teilhabehindernisse gezielt abbaut. Nur so lässt sich das Autonomiepotenzial digitaler Gesundheitsversorgung nachhaltig entfalten.

## Politisch-regulatorische Rahmenbedingungen für Autonomiegewinn

**Abb. 3:**
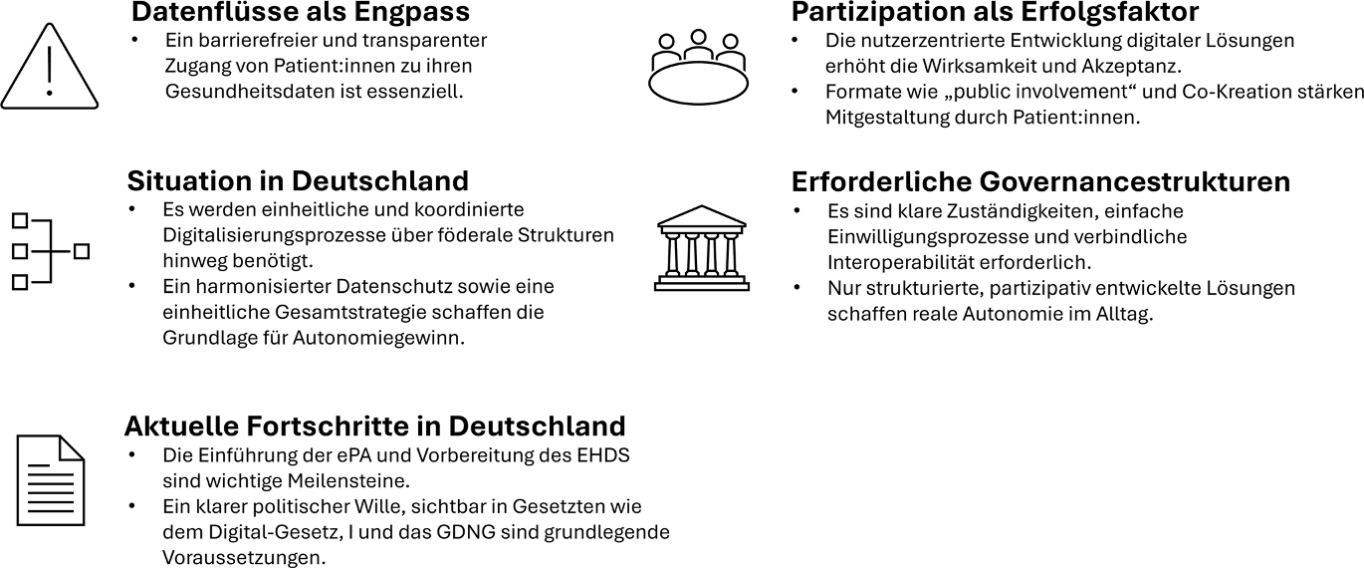
Patient:innenautonomie: systemische und regulatorische Rahmenbedingungen, *ePA* elektronische Patientenakte, *EHDS* European Health Data Space, Europäischer Gesundheitsdatenraum, *GDNG* Gesundheitsdatennutzungsgesetz. (Eigene Abbildung)

Über sozialstrukturelle Faktoren hinaus hängt die Wirksamkeit digitaler Strukturen im Gesundheitswesen zur Autonomiestärkung maßgeblich von systemischen und regulatorischen Rahmenbedingungen ab [32]. Dazu zählen gesetzliche Vorgaben, institutionelle Zuständigkeiten, Interoperabilitätsanforderungen, Datenschutzregelungen, Vergütungsmechanismen sowie geeignete technische Infrastrukturen und partizipative Mitgestaltungsmöglichkeiten. Nur wenn diese Rahmenbedingungen kohärent zusammenspielen, kann eine Versorgung entstehen, die Patient:innenautonomie aktiv ermöglicht (Abb. 3).

Ein zentraler Engpass im Aufbau autonomieförderlicher digitaler Versorgung liegt in der unzureichend geregelten Gestaltung digitaler Datenflüsse. Zwar haben Patient:innen formal ein Recht auf Einsicht in ihre Gesundheitsdaten, faktisch jedoch scheitert die Umsetzung häufig daran, dass Informationen nur fragmentiert digital vorliegen und weitergegeben werden. Oft müssen Patient:innen Befunde eigenständig übermitteln oder digitale Zugangshürden ohne Unterstützung bewältigen. Verbindliche, sektorenübergreifende Standards, klar geregelte Verantwortlichkeiten und eine kohärente Datengovernance sind dringend erforderlich [21].

Ein Blick in andere europäische Länder zeigt, dass eine umfassende Digitalisierung im Gesundheitswesen weder technisch noch rechtlich unrealistisch ist: Estland verfügt über eine interoperable nationale Gesundheitsdatenplattform mit klarem Rechtsrahmen und digitalem Zugang für Bürger:innen [37]. Auch Österreich zeigt mit der ePA, wie einheitliche technische Standards und eine sektorenübergreifende Integration zu höherer Nutzerakzeptanz führen können [38].

Während andere Länder konkrete Fortschritte bei der digitalen Transformation erzielen, gelingt es Deutschland bislang nur begrenzt, vergleichbare Strukturen umzusetzen. Der Ausbau digitaler Infrastrukturen verläuft heterogen, föderale Zuständigkeiten erschweren eine kohärente Steuerung, und die Abstimmung zwischen Politik, Selbstverwaltung und Industrie bleibt vielfach defizitär [39, 40]. Zudem wirkt die Spannung zwischen Datenschutzvorgaben und Nutzbarkeit digitaler Gesundheitsinformationen als strukturelles Hemmnis. Eine konsistente Gesamtstrategie wird benötigt, die strukturelle Defizite systematisch adressiert und das Autonomiepotenzial digitaler Versorgung gezielt einbezieht [41].

Trotz bestehender Herausforderungen zeichnen sich auch in Deutschland Fortschritte ab. Mit der verpflichtenden ePA für alle gesetzlich Versicherten und der Vorbereitung des Europäischen Gesundheitsdatenraums (EHDS) werden zentrale Weichen für Datensouveränität, sektorübergreifende und forschungskompatible Informationsflüsse gestellt. Gesetzesvorhaben wie das Digital-Gesetz (DigiG) oder das Gesundheitsdatennutzungsgesetz (GDNG) belegen die politische Bereitschaft zur Entwicklung eines modernen, datengestützten Gesundheitssystems. Die begleitende Debatte um Datenschutz, Einwilligungsmodelle und Governance eröffnet Chancen, Autonomie als Bestandteil der digitalen Transformation zu verankern. Gleichwohl bleibt die Umsetzung einiger Vorhaben fragmentiert, sodass ihr Potenzial bislang nur partiell ausgeschöpft wird [39, 40].

Auch Akzeptanz und Wirksamkeit digitaler Lösungen sind entscheidend für die digitale Transformation im Gesundheitswesen. Voraussetzung ist ihre Orientierung an den tatsächlichen Bedürfnissen der Nutzer:innen. Partizipative Formate leisten hierzu einen wichtigen Beitrag, da sie Patient:innen als Mitgestaltende einbeziehen. Verfahren wie „public involvement“, das strategische Beteiligung auf politischer oder institutioneller Ebene ermöglicht, oder Co-Kreation, bei der digitale Lösungen gemeinsam mit Betroffenen entwickelt werden, eröffnen Wege nutzerzentrierter Innovation [42].

In Deutschland gibt es erste Ansätze zur Co-Kreation, etwa in Reallaboratorien an Universitätskliniken [43], durch Patient:innenbeiräte in der Medizininformatik-Initiative [44] oder in Co-Design-Workshops einzelner Krankenkassen [45]. International sind solche Verfahren bereits stärker verankert, wie die Living Labs in den Niederlanden [46], das patient:innengeführte Modell PatientsLikeMe [47] oder die Patient:innenpanels im Swiss Personalized Health Network (SPHN, [48]) zeigen. So können digitale Technologien praxisnäher und mit Blick auf den Versorgungsalltag anschlussfähig und alltagstauglich gestaltet werden.

*Aus Patient:innenperspektive* steht fest, dass die Digitalisierung ein Motor für Gerechtigkeit sein kann, wenn sie mit den Menschen gemeinsam gestaltet wird. Patient:innenautonomie entsteht nicht durch Technik allein, sondern durch Verständlichkeit, Zugang, Vertrauen und klare Strukturen.

Für eine Stärkung der Patient:innenautonomie bedarf es einer Governancearchitektur mit klaren Verantwortlichkeiten, überprüfbaren Rechenschaftspflichten, einfachen Einwilligungsprozessen und einer verbindlichen Interoperabilitätsstrategie. Besonders wenn digitale Anwendungen partizipativ entwickelt, an Versorgungskontexte angepasst und durch klare Rahmenbedingungen begleitet werden, können sie Handlungsspielräume tatsächlich erweitern [49]. So wird Patient:innenautonomie von einem normativen Anspruch zu gelebter Realität im Versorgungsalltag.

## Fazit: Chancen nutzen, Voraussetzungen gestalten

Die Digitalisierung des Gesundheitswesens schafft neue Voraussetzungen für eine stärkere Einbindung von Patient:innen und eine Erweiterung individueller Entscheidungs- und Teilhabemöglichkeiten. Sie bietet das Potenzial, Patient:innenautonomie strukturell neu zu fundieren, etwa durch verbesserten Informationszugang, transparente Datenprozesse, personenzentrierte Versorgungsangebote und neue Formen partizipativer Mitgestaltung.

Ob das emanzipatorische Potenzial der digitalen Transformation wirksam wird, hängt nicht von der Technik allein ab. Erforderlich sind kohärente rechtliche, technische und sozialstrukturelle Rahmenbedingungen. Ebenso entscheidend sind die Stärkung der digitalen Gesundheitskompetenz von Patient:innen sowie auch die Gestaltung inklusiver Lösungen und Strukturen, die eine breite Teilhabe ermöglichen. Erst im Zusammenspiel all dieser Komponenten kann Patient:innenautonomie nicht nur postuliert, sondern im Versorgungskontext auch tatsächlich umgesetzt werden.

Digitale Angebote müssen die Diversität der Nutzer:innen berücksichtigen. Andernfalls besteht das Risiko, dass digitale Anwendungen nicht zur Stärkung, sondern zur Einschränkung von Autonomie beitragen, etwa durch intransparente Entscheidungslogiken, fragmentierte Informationsflüsse oder unzureichende Einbindung vulnerabler Gruppen. Partizipative Entwicklungsprozesse können hier den Weg zu inklusiven, nutzbaren und wirksamen Lösungen ebnen.

Auf dieser Grundlage ist eine erweiterte Perspektive notwendig, in der Patient:innen nicht weiter als passive Subjekte verstanden werden, sondern als informierte und einbezogene Akteur:innen einer lernenden, fairen und vorausschauenden Gesundheitsversorgung. Normative Leitkonzepte wie die Rechte auf Datennutzung, Dateneinsatz und Gefundenwerden bilden hierfür einen orientierenden Rahmen. Ebenso zentral ist ein zukunftsgerichtetes Verständnis von Datenschutz, das Schutzinteressen nicht in Gegensatz zur Nutzung stellt, sondern als Voraussetzung für vertrauenswürdige, zugängliche und verantwortungsvolle Datenpraktiken im Gesundheitswesen begreift.

*Aus Patient:innenperspektive* ist Digitalisierung im Gesundheitswesen nur wirksam, wenn Patient:innen aktiv und strukturell in die Entwicklung, Regulierung und Bewertung einbezogen werden. Dafür werden rechtliche, technische und soziale Rahmenbedingungen benötigt. Teilhabe darf kein Schlagwort bleiben, sondern muss institutionell verankert und methodisch gesichert sein. Digitalisierung im Gesundheitswesen darf kein Selbstzweck sein. Sie soll Patient:innen nicht nur versorgen, sondern ermächtigen, zur informierten Entscheidung, zur Teilhabe und zur Mitgestaltung. Nur so entfaltet Digitalisierung ihr emanzipatorisches Potenzial und schafft inklusive Lösungen, die echte Lebensrealitäten berücksichtigen und Patient:innen zu informierten Akteur:innen befähigen.

Um das volle Potenzial der Digitalisierung des Gesundheitssystems zur Stärkung der Patient:innenautonomie auszuschöpfen, bedarf es einer ganzheitlichen Perspektive und verantwortungsvollen Umsetzung. Wenn es gelingt, politische Rahmenbedingungen, organisatorische Strukturen und individuelle Nutzungserfahrungen in Einklang zu bringen, kann die digitale Transformation zu einem Motor für Selbstbestimmung, Transparenz und Gerechtigkeit im Gesundheitswesen werden. So entsteht ein Gesundheitssystem, das nicht nur effizient und innovativ ist, sondern auch zugänglich, gerecht und den Menschen zugewandt – und damit neue Maßstäbe für patient:innenzentrierte Versorgung setzt.
